# Antimicrobial and Dyeing Properties of Reactive Dyes with Thiazolidinon-4-one Nucleus

**DOI:** 10.1155/2014/894250

**Published:** 2014-03-04

**Authors:** Hailemichael Ayalew, Gebremedihin Reda, Tsegaye Gashaw, Neelaiah Babu, Raj Kumar Upadhyay

**Affiliations:** ^1^Department of Chemistry, College of Natural and Computational Sciences, Haramaya University, P.O. Box 276, Haramaya, Ethiopia; ^2^Department of Chemistry, College of Natural and Computational Sciences, Mekelle University, Ethiopia; ^3^Department of Chemistry, College of Natural and Computational Sciences, Assosa University, Ethiopia

## Abstract

Four imines, the condensation products of 2,4-dioxo-4-phenylbutanal with four primary amines, were condensed with mercapto acetic acid to obtain thiazolidinon-4-ones which on subsequent condensation with vanillin and isatin separately yielded eight thiazolidin-4-one derivatives. The chemical structures of the synthesized compounds were elucidated by elemental analysis, molecular weight determination, IR and ^1^H and ^13^C NMR spectral measurements. Antibacterial and antifungal properties were studied *in vitro* against two bacteria and two fungi. The dyeing potential of synthesized reactive dyes was investigated with regard to silk, wool, cotton, and polyester fabrics under hot and cold dyeing conditions.

## 1. Introduction

A small heterocyclic ring containing nitrogen and sulphur has been under investigation for pretty long time owing to their potential biological features. Among these heterocyclics, thiazolidinones having thiazol nucleus have been reported to display wide spectrum ofimportant biological activities leading to their medicinal usage such as anticancer [1], antitubercular [2], anti-HIV [3], analgesic [4], anti-inflammatory [4], ulcerogenic [5], sedative [6], antiviral [7], CNS depressant [7], hypnotic [8], antithyroidal [9], antibacterial [10, 11], and antifungal [12, 13]. Besides the pivotal role of thiazolidinones in the field of medicinal science, their many derivatives, exhibiting herbicidal [14], pesticidal [15], and insecticidal [15] properties, have significant role in agriculture. The dyeing [16–18] and ligation [19] properties of thiazolidinones have also been reported. Reactive dyes believed to form homopolar bonds with textile substrates [16, 17] with special finishing capabilities are currently an area of active research. The synthesized thiazolidinone derivatives exhibiting antimicrobial properties and possessing several auxochromic and chromophoric groups reactive with synthetic (cellulose) and natural (protein) fabrics tempted us to evaluate their dyeing potentials with silk, wool, cotton, and polyester fabrics with simultaneous antimicrobial finishing of the textiles.

## 2. Results and Discussion

**Scheme 1:**
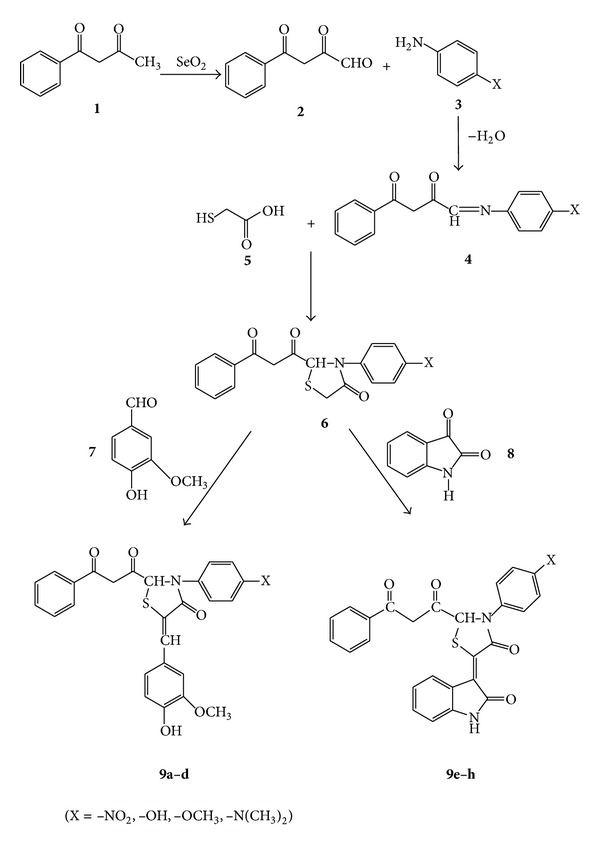
Synthetic routes to intermediates (2, 4, 6) and target compounds **9a–h**.

The synthesis of compounds **9a–h** is based on the synthetic routes shown in Scheme 1. Commercially available Benzoyl acetone (**1**) was oxidized into its glyoxal, 2,4-dioxo-4-phenylbutanal (**2**), by using selenium dioxide in ethanol. Then, the reaction of glyoxal (**2**) with substituted anilines such as p-NO_2_, p-OH, p-OCH_3,_ and p-N(CH_3_)_2_ in dry ethanol yielded 3-oxo-3-phenylpropanal azomethines (**4**) which on cyclocondensation with thioglycolic acid in dry benzene provided 2-(3-oxo-3-phenylpropanal)-3-(substituted aryl)-1-thiazolidin-4-ones. The final compounds **9a–h** were prepared by the condensation reaction of two carbonyl compounds namely vanillin and isatin in dry methanol using sodium acetate as catalyst as well as dehydrating agent.

It has been demonstrated that these reaction conditions are very useful to synthesize titled compounds with fairly high yields in relative short reaction times and easy work-up procedures. The IR, ^1^H NMR, ^13^C NMR spectral, molecular weight and elemental analysis data (Table 1) obtained are fairly consistent with the molecular formulae and structures of the synthesized compounds (Scheme 1).

### 2.1. Antimicrobial Activity

Antimicrobial tests of the synthesized thiazolidinone derivatives conducted on one Gram-negative (*Escherichia coli*) and one Gram-positive (*Staphylococcus aureus*) bacterial and selected fungi, *Aspergillus niger* and *Rhizoctonia bataticola*, revealed dose dependent activities of the new products. Compounds **9a**, **9b**, **9c,** and **9d** were found to possess significant antibacterial activity against *S. aureus* bacterium as compared with standard drug Ampicillin. Compounds **9a**, **9b**, **9c,** and **9d **exhibiting greater fungicidal activity than reference drug Bavistin against *A. niger* could be considered as highly selective for this fungus. All the new products (**9a–h**), however, showed moderate antifungal properties against *R. bataticola *(Table 2).

### 2.2. Dyeing Properties of Dyes

Since the stability and colour intensity of thiazolidinone dyes depend on pH, the concentration of sulfuric acid necessary for producing maximum colour intensity of each of the synthesized dyes has been determined spectrophotometrically as 6533 ppm, 8166 ppm, 8166 ppm, 6533 ppm, 3266 ppm, 9800 ppm, 4900 ppm, and 8166 ppm for **9a**, **9b**, **9c**, **9d**, **9e**, **9f**, **9g,** and **9h**, respectively. Dye exhaustion and fixation data (Table 3) clearly shows that all the dyes used have highest dyeing potential in cold dyeing treatment for all the four types of fibres most probably owing to the maximum reaction of dye molecules with the fibres and high stability of reaction products at room temperature. The difference in each dye colours on diverse fibres could be accounted for in terms of the different structures of their reaction products with the fibres. Fibre-wise order in dyeing potential of all the dyes in terms of fixation in both dyeing treatments is silk > wool > cotton > polyester.

### 2.3. Mechanism of Dyeing Action and Effect of Dye Auxochromes on Dye Fixation

**Figure 1:**
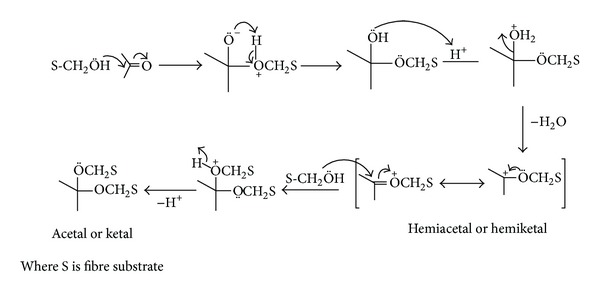
Interaction of thiazolidinone derivative dyes with cotton and polyester fibres.

All the synthesized thiazolidinone derivatives are reactive dyes with two, three, or four reactive chromophores and one or two reactive auxochromes. Primary alcoholic groups of cotton and polyester fibres interact with carbonyl groups of the dyes in presence of acid to yield unstable hemiacetals or hemiketals which on nucleophilic substitution converted to stable acetals or ketals (Figure 1).

**Figure 2:**
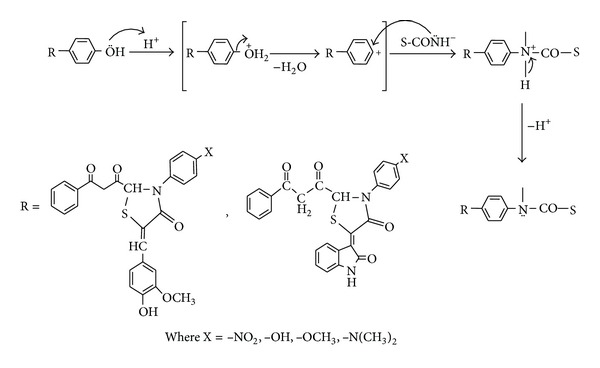
Interaction of thiazolidinone derivative dyes with silk.

Whereas, –CONH– characteristic group of the protein fibres of silk and wool interacts with enolic and/or phenolic groups of dyes in presence of acid (Figure 2).

The better fixation of all the dyes on silk and wool than cotton and polyester fibres could be accounted for in terms of more stability of the bond(s) between dyes OH group(s) and silk/wool fibre substrate owing to high reducing character of phenolic or enolic group(s) than carbonyl group (C=O) of cotton and polyester. It is worth noting that electron donor ability of auxochromic substituents plays important role in dye fixation in compounds **9(e–h)** whereas, electron donor ability of benzene ring substituents in **9(a–d)** products is insignificant in dye fixation for unknown reasons; in **9(e–h)** dye fixation order NO_2_ > OCH_3_ > OH > N(CH_3_)_2_ is opposite to electron donor ability of auxochromic substituents. This adverse effect of electron donor ability of dye auxochromes on dye fixation owes to weakening of homopolar bond between textile substrate and dye by the electronic charge transfer by the parasubstituted auxochromic group of the dye.

## 3. Experimental

### 3.1. Materials and Methods

All reagents and solvents used were either Aldrich or BDH products and used without further purification. The progress of reactions and the purity of compounds were checked by thin-layer chromatography (TLC) using Merck silica gel 60 F_254_ plates, and visualization was done in UV light (254 nm). Yields are based on purified material and were not optimized. Melting points were determined in open glass capillaries using a digital melting point apparatus (Bibby Starling LTD, ST150SA model, U.K) and are uncorrected. The ^1^H NMR and ^13^C NMR spectra were recorded on a Bruker Avance spectrometer operating at 400 MHz in DMSO/CDCl_3_ medium and chemical shifts are expressed as **δ** (ppm) with tetramethylsilane as internal standard. The spin multiplicities are reported as s (singlet), br s (broad singlet), d (doublet), t (triplet), q (quartet), and m (multiplet). The IR spectra were recorded on a FTIR-12 spectrophotometer in KBr medium. Elemental analyses were carried out on a Vario EL-III analyzer for C, H, N, and S contents. Molecular weights of the synthesized compounds were determined by Rast's method by using camphor as solvent.

### 3.2. Synthesis of Compounds

The title compounds, thiazolidinone derivatives, were synthesized by following reported procedure [16, 20] in four steps as follows.

#### 3.2.1. Synthesis of 2,4-Dioxo-4-phenylbutanal [20] (**2**)

For the synthesis of 2,4-dioxo-4-phenylbutanal [20], the reaction mixture containing 66.5 g selenium dioxide and 98.0 g of benzoylacetone (**1**) in 95% ethyl alcohol (5% water) was refluxed for ~5 h. The reaction mixture after cooling to room temperature was decanted and solvent was evaporated over water bath. The product was dissolved in ether and filtered to remove selenium. Air-dried product crystallized from ether was used for the synthesis of azomethines.

#### 3.2.2. Synthesis of 3-Oxo-3-phenylpropanoyl azomethines [20] (**4**)

For the synthesis of each azomethine [20], *p*-NO_2_ (8.6 g), *p*-OCH_3_ (7. 7 g), *p*-N(CH_3_)_2_(8.5 g), and *p*-OH (6.8 g) substituted anilines were mixed with 2,4-dioxo-4-phenylbutanal (11.0 g) (**2**) in round bottom flask containing dry ethanol and refluxed on water bath for 6 h. Concentrated reaction mixtures were cooled in an ice bath to obtain solid products. The solids obtained were crystallized from ethanol.

#### 3.2.3. Synthesis of 2-(3-Oxo-3-phenylpropanoyl)-3-(4-substituted aryl)-1-thiazolidin-4-ones [20] (**6**)

For the synthesis of 2-(3-oxo-3-phenylpropanal)-3-(substituted aryl)-1-thiazolidin-4-ones, *p*-NO_2_ (5.2 g), *p*-OCH_3_ (5.0 g), *p*-N(CH_3_)_2_ (6.0 g), and *p*-OH (4.2 g) substituted azomethines were mixed with 6.1 mL, 6.2 mL, 7.1 mL, and 5.4 mL thioglycolic acid, respectively, in dry benzene. The reaction mixtures were refluxed for 8 h and concentrated to half of their volume over water bath and then neutralized with aqueous sodium bicarbonate solution. All the contents of reaction mixtures were poured in ice cold water and filtered. The solids washed with water were dried and purified by crystallization from benzene.

#### 3.2.4. General Procedure for the Synthesis of 5-(4-Hydroxy-3-methoxybenzylidene)-2-(3-oxo-3-phenylpropanoyl)-3-(substituted aryl)-1-thiazolidin-4-ones **(9a–d)**

To synthesize title thiazolidinone dyes, vanillin (0.01 mol, 1.52 g) and each of the 2-(3-oxo-3-phenylpropanal)-3-(substituted aryl)-1-thiazolidin-4-ones (0.01 mol) were mixed together in dry methanol and fused sodium acetate (2.0 g) was added to each reaction mixture followed by refluxing for 8 h. Solids as residues of products obtained on evaporation of solvent were washed with water repeatedly and dried in air. All the products were crystallized from methanol.

#### 3.2.5. 5-(4-Hydroxy-3-methoxybenzlidene)-2-(3-oxo-3-phenylpropanoyl)-3-(p-nitrophenyl)-1-thiazolidin-4-one **(9a)**

Yield 65%, colour olive, melting point 96°C, IR (KBr, cm^−1^): *ν* 3482 (OH), 1631 (C=O), 1300 (C–N); ^1^H NMR *δ* 9.7 (s, H, OH), *δ* 3.8 (s, 2H, chain –CH_2_), 7.5 (s, 1H, CH–N), *δ* 6.6–7.5 (m, aromatic Hs), *δ* 6.6 (s, 1H, C=CH); ^13^C NMR *δ* 40, 56, 136, 158, 191.

#### 3.2.6. 5-(4-Hydroxy-3-methoxybenzlidene)-2-(3-oxo-3-phenylpropanoyl)-3-(p-hydroxylphenyl)-1-thiazolidin-4-one **(9b)**

Yield 72%, colour yellow, melting point 195°C, IR (KBr, cm^−1^): *ν* 3286 (OH), 1645 (C=O), 1331 (C–N); ^1^H NMR *δ* 9.7 (s, 2H, 2OH), *δ* 3.5 (s, 2H, chain –CH_2_), 7.5 (s, 1H, CH–N), *δ* 6.8–7.4 (m, aromatic Hs), *δ* 6.8 (s, 1H, C=CH); ^13^C NMR *δ* 20, 40, 131, 156, 187.

#### 3.2.7. 5-(4-Hydroxy-3-methoxybenzlidene)-2-(3-oxo-3-phenylpropanoyl)-3-(p-methoxyphenyl)-1-thiazolidin-4-one **(9c)**

Yield 74%, colour brown, melting point 39°C, IR (KBr, cm^−1^): *ν* 3330 (OH), 1672 (C=O), 1291 (C–N); ^1^H NMR *δ* 9.8 (s, H, OH), *δ* 4.0 (s, 2H, chain –CH_2_), 7.4 (s, 1H, CH–N), *δ* 7.0–7.4 (m, aromatic Hs), *δ* 6.9 (s, 1H, C=CH); ^13^C NMR *δ* 20, 56, 128, 157, 191.

#### 3.2.8. 5-(4-Hydroxy-3-methoxybenzlidene)-2-(3-oxo-3-phenylpropanoyl)-3-(p-N,N-dimethylamino phenyl)-1-thiazolidin-4-one (**9d**)

Yield 84%, colour saddle brown, melting point 59°C, IR (KBr, cm^−1^): *ν* 3320 (OH), 1671 (C=O), 1325 (C–N); ^1^H NMR *δ* 9.8 (s, H, OH), *δ* 4.0 (s, 2H, chain –CH_2_), 7.5 (s, 1H, CH–N), *δ* 6.8–7.5 (m, aromatic Hs), *δ* 6.7 (s, 1H, C=CH), *δ* 3.0 (s, 6H, N(CH_3_)_2_); ^13^C NMR *δ* 20, 41, 131, 164, 191.

#### 3.2.9. General Procedure for the Synthesis of 3-(2-(3-Oxo-3-phenylpropanoyl)-3-(substituted aryl)-4-oxothiazolidin-5-ylidene)indolin-2-ones **(9e–h)**

For synthesis of indigoid thiazolidinones, isatin (1.47 gm, 0.01 mol) and 2-(3-oxo-3-phenyl propanoyl)-3-(substituted aryl)-1-thiazolidin-4-ones (0.01 mol) were mixed together in dry ethanol and fused sodium acetate (5.0 g) was added to the reaction mixtures followed by refluxing for 6 h. Filtrates of reaction mixtures were evaporated on water bath and residues washed with water repeatedly were crystallized from ethanol and dried in air.

#### 3.2.10. 3-(2-(3-Oxo-3-phenylpropanoyl)-3-(p-nitro phenyl)-4-oxothiazolidin-5-ylidene)indolin-2-one **(9e)**

Yield 77%, colour sandy brown, melting point 117-118°C, IR (KBr, cm^−1^): *ν* 3480 (NH), 1735 (C=O), 1302 (CN), 697 (CS), 2874 (C–H of chain CH_2_); ^1^H NMR *δ* 2.5 (s, 2H, chain –CH_2_), 11 (s, 1H, NH), *δ* 6.5–8.5 (m, aromatic Hs); ^13^C NMR *δ* 20, 40, 130, 160, 187.

#### 3.2.11. 3-(2-(3-Oxo-3-phenylpropanoyl)-3-(p-hydroxyl phenyl)-4-oxothiazolidin-5-ylidene)indolin-2-one **(9f)**

Yield 70%, colour peru, melting point 164-165°C, IR (KBr, cm^−1^): *ν* 3495 (NH), 3195 (OH), 1730 (C=O), 1331 (CN), 673 (CS), 2809 (C–H of chain CH_2_); ^1^H NMR *δ* 5.6 (s, H, OH), *δ* 2.5 (s, 2H, chain –CH_2_), 9.7 (s, 1H, NH), *δ* 6.5–8.0 (m, aromatic Hs); ^13^C NMR *δ* 20, 40, 140, 160, 187.

#### 3.2.12. 3-(2-(3-Oxo-3-phenylpropanoyl)-3-(p-methoxy phenyl)-4-oxothiazolidin-5-ylidene)indolin-2-one **(9g)**

Yield 76%, colour orange, melting point 64–65°C, IR (KBr, cm^−1^): *ν* 3437 (NH), 1728 (C=O), 1332 (CN), 694 (CS), 2836 (C-H of chain CH_2_); ^1^H NMR *δ* 2.0 (s, 2H, chain -CH_2_), 8.5 (s, 1H, NH), *δ* 6.5–8.0 (m, aromatic Hs), *δ* 2.5 (s, 3H, OCH_3_); ^13^C NMR *δ* 23, 60, 134, 152, 174.

#### 3.2.13. 3-(2-(3-Oxo-3-phenylpropanoyl)-3-(p-N,N-dimethylaminophenyl)-4-oxothiazolidin-5-ylidene)indolin-2-one **(9h)**

Yield 87%, colour dim gray, melting point 97-98°C, IR (KBr, cm^−1^): *ν* 3461 (NH), 1730 (C=O), 1328 (CN), 694 (CS), 2924 (C–H of chain CH_2_); ^1^H NMR *δ* 2.3 (s, 2H, chain –CH_2_), 7.9 (s, 1H, NH), *δ* 6.0–7.7 (m, aromatic Hs), *δ* 1.3 (s, 6H, N(CH_3_)_2_); ^13^C NMR *δ* 22, 41, 130, 185.

### 3.3. Antimicrobial Studies

The synthesized compounds were tested *in vitro *for their antimicrobial activities by using the paper disc diffusion technique against two important bacteria, *Escherichia coli *and *Staphylococcus aureus, *using Muller Hinton agar (MHA) medium and antifungal activity against two common fungi, *Aspergillus niger *and *Rhizoctonia bataticola, *using potato dextrose agar (PDA) medium. Ampicillin, a standard drug, was used as reference in bactericidal and Bavistin, a standard fungicide, was used as reference in fungicidal studies. From inhibition zone data antimicrobial activities of the compounds were critically examined.

### 3.4. Preparation of Inoculums

The test bacterial strains, *Escherichia coli *(Gram-negative) and *Staphylococcus aureus *(Gram-positive), were transferred from the stock cultures and streaked on MHA plate and incubated for 24 h at 37°C. Then, the bacteria were transferred using inoculating loop to autoclaved MHA that was cooled to about 45°C in a water bath and mixed by gently swirling the flasks. The medium was then poured to sterilized Petri dishes, allowed to solidify, and used for the biotest. For test fungi, mycelia plugs from stock cultures were transferred to PDA plates and incubated for 6 days. Then, spores of the test fungi were harvested by washing the surface of the colony using 10 mL sterile distilled water and transferred to 50 mL autoclaved PDA cooled to about 45°C in a water bath. The media containing spore suspension were poured to sterilized plates, allowed to solidify, and were used for the disc diffusion bioassay.

### 3.5. Preparation of Sample Solutions

The synthesized thiazolidinone derivatives were prepared by dissolving 10 mg in 2 mL of dimethylsulphoxide and used for testing.

### 3.6. Testing for Antifungal Activity

Filter paper discs of 6 mm diameter placed in a beaker were sterilized in an oven at 180°C for 1 h. Then 10 *μ*L and 20 *μ*L of solution of compounds were pipetted to the discs in three replications. After allowing the solvent to evaporate, the paper discs impregnated with the samples were then transferred with sterilized forceps to PDA seeded with spore suspension of test fungi as described above. The Petri dishes were incubated at 25°C for 2-3 days. The entire test was performed in triplicate. The antifungal activity was evaluated by measuring the zone of inhibition against the test organism.

### 3.7. Testing for Antibacterial Activity

Similar procedures done for antifungal activity test were followed in antibacterial studies except the paper discs were transferred to nutrient agar plate seeded with bacteria and incubated at 37°C for *Staphylococcus aureus *and 28°C for *Escherichia coli *for 25 h.

### 3.8. Dyeing Studies

Solutions of thiazolidinone dyes were prepared by dissolving 10 mg of each compound directly in MeOH-H_2_O (1 : 1 v/v) for *λ*_max⁡_ determination. At difference of 2 nm optical density was measured and graphs were plotted in wavelength versus optical density of solution to determine *λ*_max⁡_ of each sample.

For quantitative studies standard solutions of thiazolidinones were prepared by dissolving known quantities of samples in known volumes of the solvent, MeOH-H_2_O (1 : 1 v/v).

For finding out necessary H_2_SO_4_ concentration for producing maximum colour intensity of each dye, six equiconcentrated solutions of each dye sample were prepared in MeOH-H_2_O (1 : 1 v/v) solvent containing different quantities of acid and optical densities were measured at *λ*_max⁡_ of each dye. From the highest value of optical density of the dye at its *λ*_max⁡_ requisite concentration of acid necessary for producing maximum colour was determined. For the preparation of calibration curves, optical density at *λ*_max⁡_ of each dye solution at different concentrations containing requisite quantity of acid was measured.

### 3.9. Dying and Postdyeing Treatment

Two of the four prewashed white cloth pieces of 8 cm × 8 cm size of silk, wool, cotton, and polyester were dipped in 10 mL of 500 ppm dye solution in each beaker at room temperature and 80 ± 5°C containing necessary concentration of H_2_SO_4_ separately and were left for 1 h, squeezed, and dried in sunlight. Then two pieces of each fibre, dyed by each dye using cold and hot methods, were washed with water and dried in sunlight.

### 3.10. Determination of Total Dye Exhaustion and Fixation of Dye

For determination of total dye exhaustion, one piece of each fibre dyed by each method was squeezed and optical density of extracted solution of each dye was measured to find out concentration from its calibration curve prepared under similar conditions of temperature and solvent; from the concentration of extract total dye exhaustion was calculated. Dyed dry clothes were dipped in 20 mL water and were left for 30 minutes and then squeezed and optical density of the extract was measured and concentration was determined from calibration curve to know the fixation of dye on each fibre. Dye exhaustion (%*E*) and fixation (%*F*) were calculated using the following equations [21]:

(1) $$\begin{array}{c} \%E=\frac{\left(D_{o}-D_{f}\right)}{D_{o}}\times 100,\\ \%F=\frac{\left(D_{o}-D_{f}-D_{e}\right)}{D_{o}-D_{f}}\times 100, \end{array}$$

where *D*_0_, *D*_*f*_, and *D*_*e*_ are concentration of dye before and after dyeing and amount of extracted dyes, respectively.

## 4. Conclusion

Based on our experimental findings, all the new triaryl substituted derivatives containing thiazolidinone moiety exhibiting excellent bactericidal and fungicidal and dyeing potentials could be proposed for dyeing and antimicrobial finishing for silk, wool, cotton, and polyester fabrics.

## Figures and Tables

**Table 1:** Physicochemical properties of dyes.

Dye	m.f	*p*-substitu ents	Yield (%)	m.p (°C)	m.w., calcd. (found)	Elemental analysis (%): calcd. (found)				*λ*_ max_ (nm)
						C	H	N	S	
**9a**	C_26_H_20_N_2_O_7_S	*p*-NO_2_	65	96	504 (492.37)	61.90 (60.84)	3.97 (3.85)	5.56 (5.94)	6.35 (6.41)	384
**9b**	C_26_H_21_NO_6_S	*p*-OH	72	195	475 (470.55)	65.68 (65.07)	4.42 (4.41)	2.95 (3.29)	6.74 (6.27)	356
**9c**	C_27_H_23_NO_6_S	*p*-OCH_3_	74	39	489 (492.37)	66.26 (65.67)	4.70 (4.71)	2.86 (2.61)	6.54 (6.95)	310
**9d**	C_22_H_26_N_2_O_5_S	*p*-N(CH_3_)	84	59	502 (492.37)	66.93 (66.16)	5.18 (4.62)	5.58 (5.29)	6.37 (6.00)	312
**9e**	C_26_H_17_N_3_O_6_S	*p*-NO_2_	77	118	499 (492.5)	65.52 (62.21)	3.40 (3.90)	8.41 (8.53)	6.41 (6.20)	380
**9f**	C_26_H_18_N_2_O_5_S	*p*-OH	70	165	470 (460.6)	66.38 (65.92)	3.82 (4.40)	5.95 (6.22)	6.80 (6.70)	360
**9g**	C_27_H_20_N_2_O_5_S	*p*-OCH_3_	76	65	484 (476.98)	66.94 (65.04)	4.13 (4.12)	5.78 (5.70)	6.61 (6.40)	350
**9h**	C_28_H_23_N_3_O_4_S	*p*-N(CH_3_)	87	98	497 (484.57)	66.38 (65.72)	4.62 (4.80)	8.45 (8.17)	6.43 (6.45)	320

**Table 2:** Zone of bacterial and fungal growth inhibition (mm) for compounds **9a**–**h**.

S. no	Compounds	Zone of inhibition							
		Bacteria				Fungi			
		*E. coli *		*S. aureus *		*A. niger *		*R. bataticola *	
		10 µL	20 µL	10 µL	20 µL	10 µL	20 µL	10 µL	20 µL
1	**9a**	11	13	29	31	27	29	19	23
2	**9b**	11	12	30	31	24	26	18	24
3	**9c**	10	11	30	31	27	29	11	13
4	**9d**	10	11	28	30	27	29	13	18
5	**9e**	11	12	10	14	12	13	10	15
6	**9f**	13	14	10	13	13	15	14	16
7	**9g**	08	10	10	12	11	13	15	16
8	**9h**	10	12	08	12	13	14	12	14
9	Ampicillin	20	22	38	40	—	—	—	—
10	Bavistin	—	—	—	—	19	21	21	24
11	DMSO	—	—	—	—	—	—	—	—

Key: All results are mean of three replications.

**Table 3:** Fibre colour, exhaustion and fixation data of dyes.

Dye	Dyeing method	Dye colour				Exhaustion (%)				Fixation (%)				Necessary H_2_SO_4_ conc. (ppm)
		Silk	Wool	Cotton	Polyester	Silk	Wool	Cotton	Polyester	Silk	Wool	Cotton	Polyester	
**9a**	Cold	Gold	—	Burly wood	Light yellow	79	—	68	73	94	—	71	80	6533
	Hot	Gold		Burly wood	Light yellow	78	—	65	61	77	—	51	67	6533
**9b**	Cold	Yellow	—	Light yellow	Yellow	80	—	78	72	99	—	97	95	8166
	Hot	Light yellow		Light yellow	Light yellow	77	—	70	63	98	—	96	94	8166
**9c**	Cold	Burly wood	—	Light yellow	Light yellow	78	—	69	72	96	—	89	89	8166
	Hot	Burly wood		Light yellow	Light yellow	75	—	68	62	87	—	84	74	8166
**9d**	Cold	Bisque	—	Light yellow	Pale yellow	35	—	36	35	89	—	77	61	6533
	Hot	Bisque		Light yellow	Pale yellow	35	—	35	35	80	—	54	45	6533
**9e**	Cold	Khaki	Burly wood	Burly wood	Tan	87	85	80	78	90	88	83	80	3266
	Hot	Khaki	Burly wood	Burly wood	Tan	77	75	71	60	78	77	75	67	3266
**9f**	Cold	Pale golden	Sea shell	Tan	Ghost white	75	70	68	65	78	76	72	69	9800
	Hot	Pale golden	Sea shell	Tan	Ghost white	72	65	62	60	75	68	65	60	9800
**9g**	Cold	Golden rod	Navajo white	Burly wood	Pale golden	84	82	78	75	86	85	79	76	4900
	Hot	Golden rod	Navajo white	Burly wood	Pale golden	74	71	69	64	76	75	70	68	4900
**9h**	Cold	Alice blue	Old lace	Light gray	Ivory	73	67	63	61	77	72	65	64	8166
	Hot	Alice blue	Old lace	Light gray	Ivory	70	62	58	50	71	64	62	56	8166
